# Therapeutic Potential of Curcumin, a Bioactive Compound of Turmeric, in Prevention of Streptozotocin-Induced Diabetes through the Modulation of Oxidative Stress and Inflammation

**DOI:** 10.3390/molecules29010128

**Published:** 2023-12-25

**Authors:** Abdullah Khalid Alsulaim, Turki Hussain Almutaz, Abdulaziz Ahmed Albati, Arshad Husain Rahmani

**Affiliations:** Department of Medical Laboratories, College of Applied Medical Sciences, Qassim University, Buraydah 51452, Saudi Arabia; abdullahalsulaim9@gmail.com (A.K.A.); tur.ka4@hotmail.com (T.H.A.); a.a.albati@hotmail.com (A.A.A.)

**Keywords:** diabetes mellitus, curcumin, inflammation, oxidative stress

## Abstract

This study evaluates the anti-diabetic potential and underlying mechanisms of curcumin in streptozotocin (STZ)-induced type 2 diabetes mellitus (T2DM) rats. The rats were randomly divided into four groups: normal control, negative control (diabetic group), diabetic group receiving glibenclamide (positive control group), and curcumin plus STZ (treatment group). The anti-diabetic activities of curcumin were examined at a dose of 50 mg/kg body weight through physiological, biochemical, and histopathological analysis. Compared to the normal control group rats, elevated levels of glucose, creatinine, urea, triglycerides (TG), and total cholesterol (TC) and low levels of insulin were found in the negative control rats. Curcumin treatment showed a significant decrease in these parameters and an increase in insulin level as compared to negative control rats. In negative control rats, a reduced level of antioxidant enzymes and an increased level of lipid peroxidation and inflammatory marker levels were noticed. Oral administration of curcumin significantly ameliorated such changes. From histopathological findings, it was noted that diabetic rats showed changes in the kidney tissue architecture, including the infiltration of inflammatory cells, congestion, and fibrosis, while oral administration of curcumin significantly reduced these changes. Expression of IL-6 and TNF-α protein was high in diabetic rats as compared to the curcumin treatment groups. Hence, based on biochemical and histopathological findings, this study delivers a scientific suggestion that curcumin could be a suitable remedy in the management of diabetes mellitus.

## 1. Introduction

Higher glucose levels and abnormalities in insulin production characterize diabetes mellitus (DM), or insulin resistance, and some people can have both [1]. Prolonged DM usually leads to various complications, including cardiovascular disease, chronic kidney disease, diabetic ketoacidosis, and a hyperosmolar hyperglycemic state [2]. Moreover, lipid abnormalities are major players in DM due to insulin resistance or metabolic changes that disrupt key enzymes and lipid metabolic pathways [3]. As per the International Diabetes Federation, it is projected that the number of diabetes patients will increase to approximately 10% (578 million) by 2030 and 10.9% (700 million) by 2045 [4]. Thus, the regulation of blood glucose levels is vital for preventing diabetic complications as well as improving the health of diabetic patients [5]. The current modes of treatment for DM may be effective, but in parallel also cause some adverse complications. 

The wide and innumerable number of natural compounds from animals, plants, microorganisms, fungi, and other natural resources delivers a rich and inimitable source in the search for new drugs [6]. In addition, in traditional medicine, several medicinal plants with hypoglycemic properties have been used for treating DM [7].

**Figure 1:**
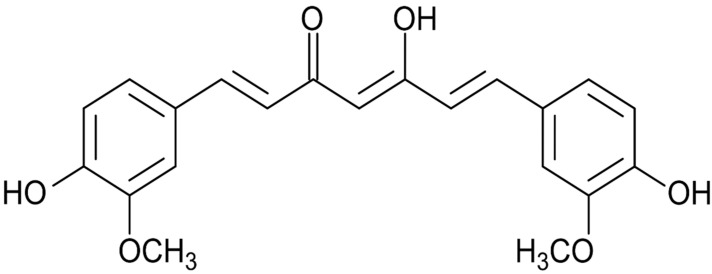
Chemical structure of curcumin.

In this regard, curcumin, a yellow-colored compound, is produced by plants of *Curcuma longa* species, and it is chemically known as 1,7-bis(4-hydroxy-3-methoxyphenyl)-1, 6-heptadiene-3, 5-dione (Figure 1). It possesses antioxidant, anti-inflammatory, anti-tumor, and other biological activities [8].

Curcumin is capable of exercising its antioxidant action via scavenging a variety of hydrogen peroxide and nitric oxide (NO) radicals and reactive oxygen species (ROS) as superoxide radicals and by preventing lipid peroxidation [9]. Moreover, several in vitro as well as in vivo studies have described that curcumin has potential for treating numerous inflammatory diseases [10,11,12,13]. A recent study based on the nephroprotective effect of curcumin in STZ-induced DM was performed. The study revealed that curcumin significantly reduced blood urea nitrogen, serum levels of urea, and creatinine and simultaneously reduced albumin/protein urea and increased creatinine clearance. Further, it also prevented damage to renal tubules and the thickness of the basement membrane [14]. In addition, STZ induction caused increased hepatic damage linked to the serum levels of ALT, ALP, and LDH, increased the production of NO, increased ROS generation and lipid peroxidation, and reduced antioxidant enzyme levels. However, curcumin treatment efficiently counters diabetes-induced oxidative-stress-mediated hepatic damage [15]. Another finding reported that curcumin improved the survival as well as the function of islet cells, with reduced cell apoptosis in the islet of Langerhans and increased insulin secretion in the STZ-induced diabetic model [16]. 

In this study, the therapeutic potential of curcumin on streptozotocin (STZ)-induced kidney injury in rats was evaluated via inflammation, the lipid profile, oxidative stress, and other biochemical parameters. Moreover, kidney tissue architecture was evaluated via hematoxylin and eosin, fibrosis by Masson trichrome and Sirius red, and inflammatory protein expression by immunohistochemistry staining.

## 2. Results

### 2.1. Role of Curcumin on Oral Glucose Tolerance Tests (OGTTs)

**Figure 2:**
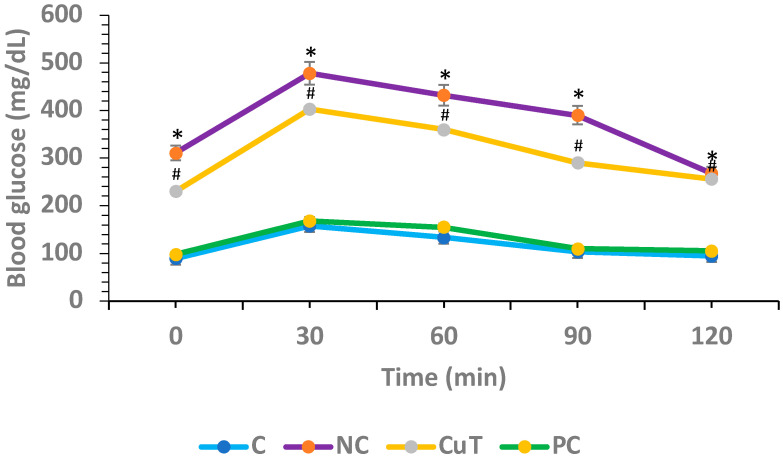
Role of curcumin on oral glucose tolerance tests (OGTTs) was evaluated. Mean ± SEM was used to describe the findings: * *p* < 0.05 (significant variance in OGTTs between negative control (NC) and normal control (C)); # *p* < 0.05 (significant difference between NC and curcumin treatment (CuT)).

Hyperglycemia is the most significant indication of diabetes, and OGTTs were performed to measure the hyperglycemic activity in different experimental groups of rats. In the control group, glucose levels were estimated at 0, 30, 60, 90, and 120 min as (89.4 ± 4.2, 157.6 ± 6.8, 133.2 ± 7.3, 103.4 ± 5.6, and 94.8 ± 4.7 mg/dL), and negative control rats revealed higher blood glucose levels at same time intervals as (310.8 ± 7.9, 478.6 ± 9.8, 431.8 ± 8.4, 390.2 ± 5.8, and 268.4 ± 4.9 mg/dL) (*p* < 0.05). The treatment of diabetic rats with curcumin showed a noteworthy reduction in glucose levels (230.4 ± 4.7, 403.7 ± 5.9, 360.5 ± 7.2, 290.5 ± 5.2, and 256.7 ± 3.5 mg/dL) when compared to the negative control rats at the same time intervals (*p* < 0.05) (Figure 2).

### 2.2. Role of Curcumin on Glucose and Insulin Levels

**Figure 3:**
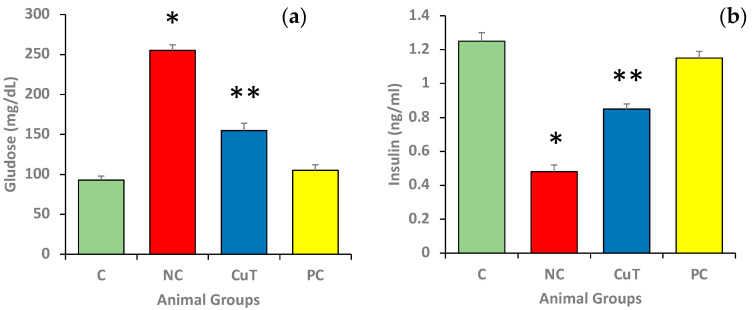
The levels of (**a**) glucose and (**b**) insulin were measured in different experimental groups of rats (a total of 8 rats in each group). Mean ± SEM was used to define the findings: * *p* < 0.05 (significant variance in b.w. (final) between negative control (NC) and normal control (C)); ** *p* < 0.05 (significant difference between NC and curcumin treatment (CuT)).

Figure 3 shows the levels of fasting glucose and insulin in different experimental animals. The fasting glucose levels were found to be significantly higher in the negative control rats as compared to control group rats (255 ± 7 mg/dL vs. 93 ± 5 mg/dL) (*p* < 0.05) (Figure 3a), whereas insulin levels were significantly lowered in the negative control animals in comparison with control group rats (0.48 ± 0.04 ng/mL vs. 1.25 ± 0.05 ng/mL) (Figure 3b). The animals treated with curcumin exhibited a substantial reduction in fasting glucose (155 ± 9 mg/dL) and increased insulin levels (0.85 ± 0.03 ng/mL) when compared to negative control group rats (*p* < 0.05). The glibenclamide (positive control) displayed the same potential in the reduction in FBG and enhancement of insulin, and the values were near those of the control group.

### 2.3. Effect of Curcumin on Lipid Profile

**Figure 4:**
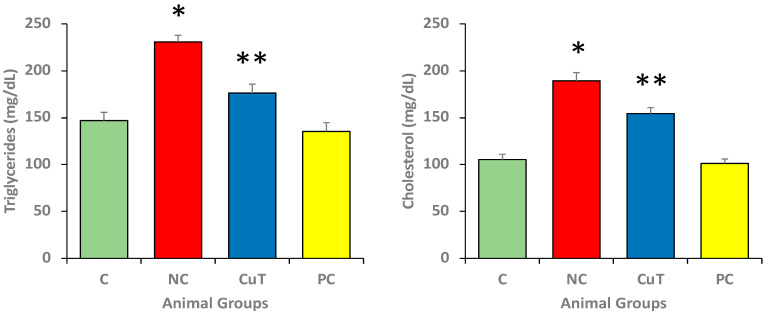
The serum levels of triglycerides (TG) and total cholesterol (TC) were evaluated in rats (8 rats in each group). Mean ± SEM was used to define the findings: * *p* < 0.05 (significant variance in b.w. (final) between negative control (NC) and normal control (C)); ** *p* < 0.05 (significant difference between NC and curcumin treatment (CuT)).

The triglycerides (TGs) and total cholesterol (TC) serum levels were evaluated in different experimental groups. Figure 4 shows that the serum levels of cholesterol (189.37 ± 8.7 mg/dL) and TGs (230.7 ± 7.2 mg/dL) were increased in negative control rats in comparison with normal control rats (105.34 ± 5.7 mg/dL and 146.9 ± 8.7 mg/dL) (*p* < 0.05). The treatment with curcumin significantly (*p* < 0.05) decreased the levels of cholesterol (154.45 ± 6.4 mg/dL) and TGs (176.3 ± 9.5 mg/dL) (*p* < 0.05). These findings identified abnormalities in lipid metabolism in diabetic animals.

### 2.4. Effect of Curcumin on Creatinine and Urea Levels

**Figure 5:**
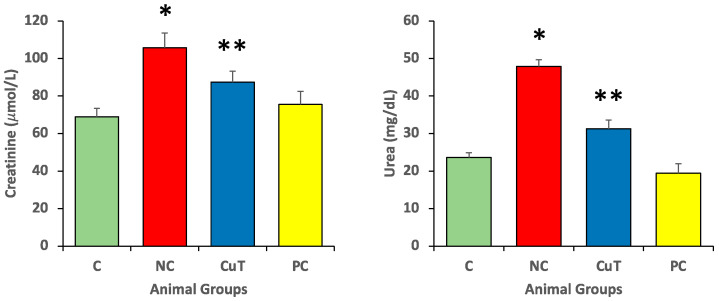
The serum levels of creatinine and total urea were evaluated in rats (a total of 8 rats in each group). Mean ± SEM was used to define the findings: * *p* < 0.05 (significant variance in b.w. (final) between negative control (NC) and normal control (C)); ** *p* < 0.05 (significant difference between NC and curcumin treatment (CuT)).

The creatinine and urea serum levels were evaluated in experimental animals in each group. Negative control group rats exhibited increased creatinine (105.7 ± 7.8 µmol/L) and urea levels (47.9 ± 1.8 mg/dL) (*p* < 0.05). Creatinine and urea levels of the STZ-induced diabetic rats (negative control) treated with curcumin (50 mg/kg) returned to normal levels (87.39 ± 5.8 µmol/L and 31.3 ± 2.3 mg/dL) (*p* < 0.05) (Figure 5). 

### 2.5. Effect of Curcumin Treatment on Oxidative Stress Level

**Figure 6:**
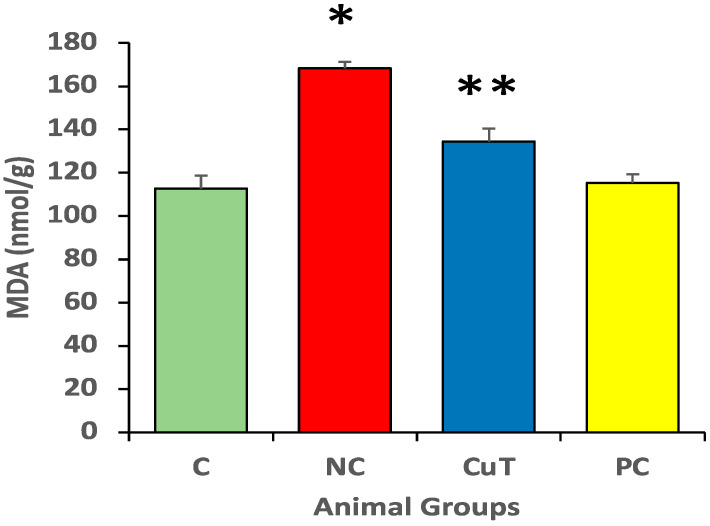
The MDA levels were evaluated in rats (a total of 8 rats in each group). Mean ± SEM was used to define the findings: * *p* < 0.05 (significant variance in b.w. (final) between negative control (NC) and normal control (C)); ** *p* < 0.05 (significant difference between NC and curcumin treatment (CuT)).

Negative control group rats showed a rise in MDA levels when compared to normal control group rats (168.3 ± 3 nmol/g vs. 112.7 ± 6 nmol/g). Figure 6 shows that treatment with curcumin (50 mg/kg body weight) significantly (*p* < 0.05) decreased MDA levels in STZ-induced negative control rats (134.4 ± 6 nmol/g) (Figure 6).

**Figure 7:**
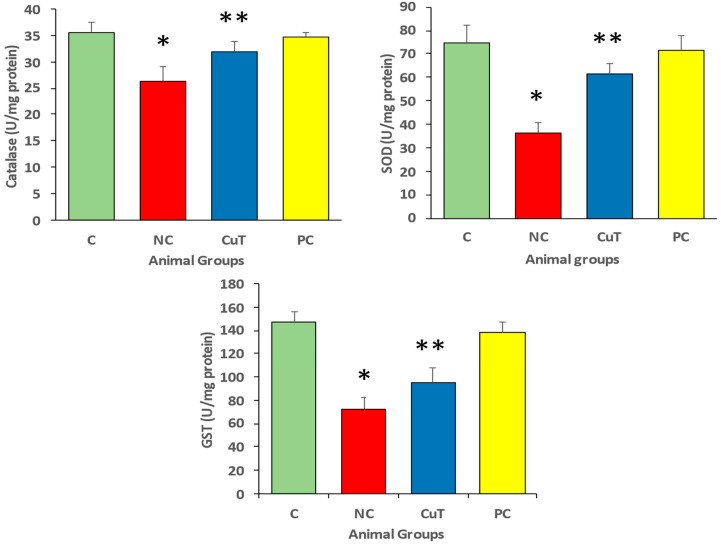
The measurement of antioxidant enzyme levels in rats (a total of 8 rats in each group). Mean ± SEM was used to define the findings: * *p* < 0.05 (significant variance in b.w. (final) between negative control (NC) and normal control (C)); ** *p* < 0.05 (significant difference between NC and curcumin treatment (CuT)).

Furthermore, the results showed that negative control rats displayed a decline in antioxidant enzyme (CAT, 26.2 ± 3 vs. 35.5 ± 2 U/mg protein; GST, 71.9 ± 10 vs. 146.6 ± 9.2 U/mg protein; and SOD, 36.5 ± 4 vs. 74.9 ± 7 U/mg protein) levels as compared to normal control groups (Figure 7). As compared to the STZ-induced negative control rats, curcumin enhances the reduced antioxidant enzymes CAT (31.8 ± 2.1 U/mg protein), GST (95.5 ± 12.2 U/mg protein), and SOD (61.7 ± 4.1 U/mg protein). This suggests that curcumin reduced oxidative stress through the enhancement of antioxidant enzymes and the reduction in lipid peroxidation.

### 2.6. Effect of Curcumin Treatments on Inflammatory Marker Level

**Figure 8:**
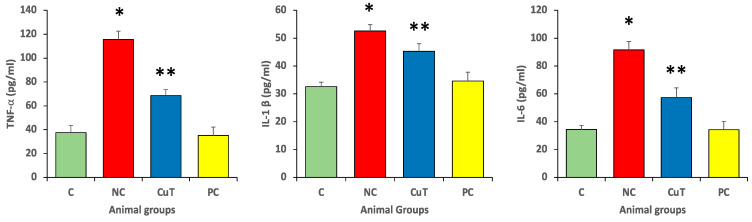
Cytokine levels were measured in rats (a total of 8 rats in each group). Mean ± SEM was used to define the findings: * *p* < 0.05 (significant variance in b.w. (final) between negative control (NC) and normal control (C)); ** *p* < 0.05 (significant difference between NC and curcumin treatment (CuT)).

Negative control rats showed a substantial elevation (*p* < 0.05) in levels of cytokines such as TNF-α (115.5 ± 6.2 vs. 37.6 ± 6.3 pg/mL), IL-6 (91.6 ± 6.2 vs. 34.43 ± 3.0 pg/mL), and IL-1β (52.6 ± 2.3 vs. 32.3 ± 1.6 pg/mL) as compared to the control group. Figure 8 shows that treatment with curcumin (50 mg/kg b.w.) suggestively (*p* < 0.05) decreased these cytokine levels (TNF-α, 68.8 ± 5.1 pg/mL; IL-6, 57.4 ± 7.2 pg/mL; and IL-1β, 45.3 ± 2.7 pg/mL) in the STZ-induced negative control group. These findings indicate that curcumin has a role in diabetes management through its anti-inflammatory properties.

### 2.7. Effect of Curcumin Treatments on Renal Tissue Architecture

**Figure 9:**
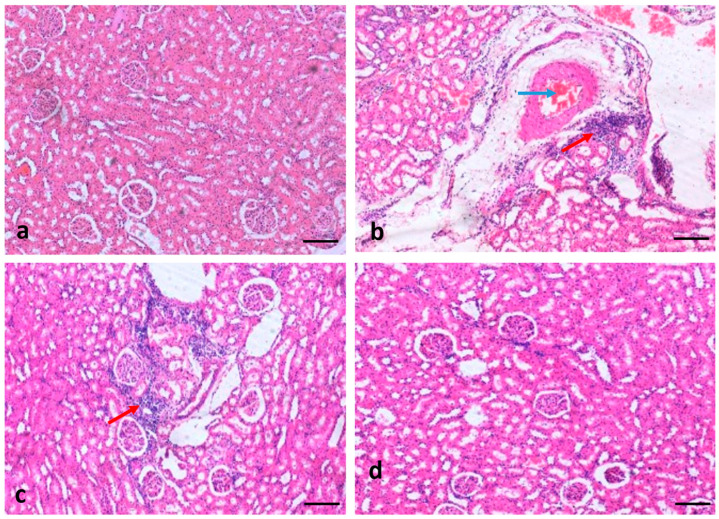
Renal tissue architecture of different experimental groups of rats: (**a**) normal control group; (**b**) STZ-induced negative control group rats; (**c**) STZ treated with curcumin (50 mg/kg body weight); (**d**) STZ treated with glibenclamide (positive control). Original magnification: 100×; scale bar: 50 μm. The red arrow shows inflammatory cells and the blue arrow shows congestion.

The hematoxylin and eosin (H&E) staining of the renal tissue of normal-group rats showed normal renal tissue architecture. Compared to the control group, negative control group rats showed various changes characterized by inflammatory cell infiltration, congestion, and fibrosis. However, the administration of curcumin in negative control group rats (50 mg/kg body weight) considerably reduced the renal tissue change. Glibenclamide-administered (positive control) rats showed normal tissue architecture (Figure 9).

### 2.8. Effect of Curcumin Treatments on Renal Fibrosis

**Figure 10:**
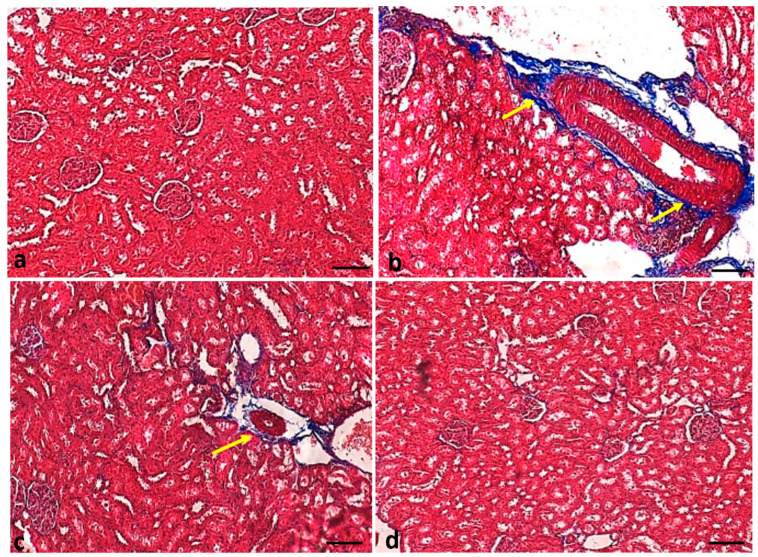
Renal tissue fibrosis in different experimental groups of rats: (**a**) normal control group; (**b**) STZ-induced diabetic group; (**c**) STZ treated with curcumin (50 mg/kg body weight; (**d**) STZ treated with glibenclamide. Original magnification: 100×; scale bar: 50 μm. Arrow (yellow) showing collagen fiber deposition.

Renal fibrosis was measured in all experimental groups using Masson trichrome and Sirius red staining to check the effects of curcumin. The Masson trichrome staining of the renal tissue of normal-group rats showed normal collagen fiber. As compared to the control group, the negative control rats showed thick collagen fiber (stained blue). However, diabetic animals treated with curcumin showed reduced fibrosis. Glibenclamide-administered (positive control) rats showed a similar result as that shown in the control group (Figure 10).

**Figure 11:**
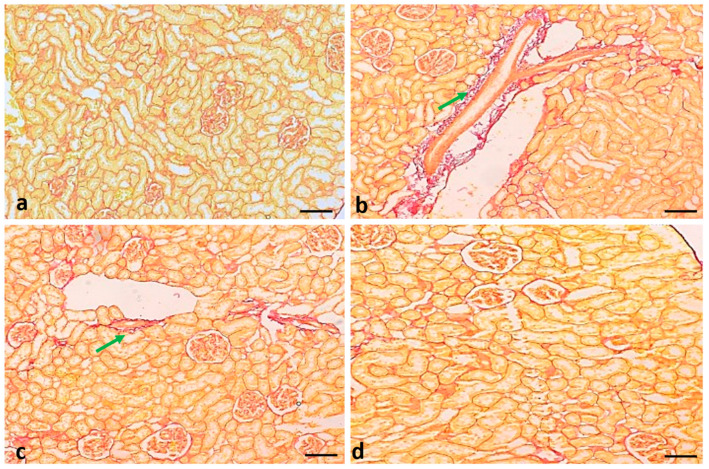
Renal tissue fibrosis in different experimental groups of rats: (**a**) normal control group; (**b**) STZ-induced negative control group; (**c**) STZ treated with curcumin (50 mg/kg body weight); (**d**) STZ treated with glibenclamide (positive control). Original magnification: 100×; scale bar: 50 μm. Arrow (green) showing fiber deposition.

The Sirius red staining of the renal tissue of normal-group rats showed normal collagen fiber. As compared to the control group, negative control rats showed more fiber (stained red). The negative control group treated with curcumin showed significantly reduced fibrosis. (Figure 11).

### 2.9. Effect of Curcumin Treatments on IL-6 Protein Expression

**Figure 12:**
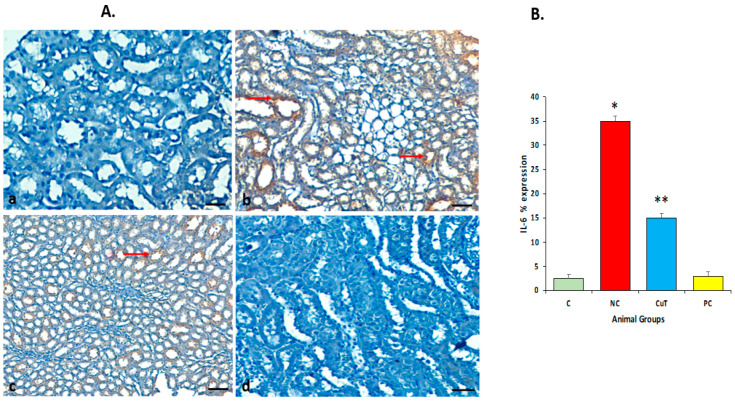
(**A**) Expression of IL-6 protein in different experimental groups of rats: (**a**) normal control group (showed no expression); (**b**) STZ-induced negative control group (showed high expression); (**c**) STZ treated with curcumin (50 mg/kg body weight) (showed less expression); (**d**) STZ treated with glibenclamide (positive control, showed no expression). Original magnification: 100×; scale bar: 50 μm. Arrow (red) showing cytoplasmic positivity. (**B**) Graph showing IL-6 expression in different experimental groups: * *p* < 0.05 (significant variance in b.w. between negative control (NC) and normal control (C)); ** *p* < 0.05 (significant difference between NC and curcumin treatment (CuT)).

Immunohistochemistry (IHC) staining was performed to evaluate the expression pattern of the IL-6 protein. The staining of renal tissue of normal group rats showed no expression of the IL-6 protein. Negative control group rats showed significant upregulation or high expression (stained brown color) (*p* < 0.05) of IL-6 in renal cells as compared to the control group, whereas negative control group rats treated with curcumin showed significantly reduced or downregulated (*p* < 0.05) expression of the IL-6 protein as compared to negative control group rats. The glibenclamide-administered (positive control) group showed a similar result (no expression) to that in the control group (*p* > 0.05) (Figure 12A,B).

### 2.10. Effect of Curcumin Treatments on TNF-α Protein Expression

**Figure 13:**
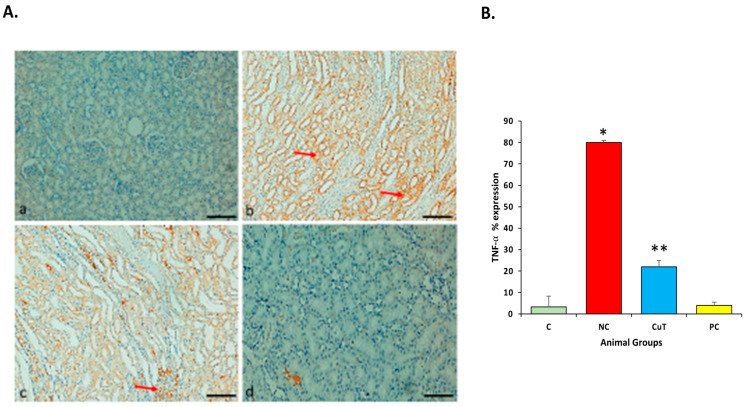
(**A**) Expression of TNF-α protein in different experimental groups of rats: (**a**) normal control group (showed no expression); (**b**) STZ-induced negative control group (showed high expression); (**c**) STZ treated with curcumin showed decreased expression; (**d**) STZ treated with glibenclamide (positive control, showed no expression). Original magnification: 100×; scale bar: 50 μm. Arrow (red) showing cytoplasmic positivity. (**B**) Graph showing TNF-α expression in different experimental groups: * *p* < 0.05 (significant variance in b.w. between negative control (NC) and normal control (C)); ** *p* < 0.05 (significant difference between NC and curcumin treatment (CuT)).

The staining of the renal tissue of normal-group rats showed no expression of the TNF-α protein. Negative control rats showed significant upregulation (stained brown color) of this protein in renal cells as compared to the control group (*p* < 0.05), whereas negative control group rats treated with curcumin showed significantly reduced or downregulated (*p* < 0.05) expression of the TNF-α protein as compared to negative control group rats (*p* < 0.05) (Figure 13A,B). The glibenclamide-administered (positive control) group did not show any expression compared to the control group rats (*p* > 0.05) (Figure 13A,B).

## 3. Discussion

Higher glucose levels and abnormalities in insulin production or action [1] commonly describe diabetes mellitus (DM). Numerous anti-diabetic drugs and synthetic inhibitors for hyperglycemia are accessible that decrease the development of diabetic complications; however, several adverse effects have been noticed in parallel [17]. Thus, natural anti-diabetic drugs or medicinal plant compounds may deliver an auspicious therapeutic remedy to prevent diabetes and its complications. 

STZ damages insulin-producing beta cells within the pancreas, and therefore it is commonly used to induce diabetes in laboratory animals [18,19]. In this study, a diabetes model was used to investigate the protective effect of curcumin against renal pathogenesis in diabetic rats. 

In the current study, it was noticed that fasting glucose levels (FBG) were found to be significantly higher and insulin levels significantly lower in the type 2 diabetic mellitus animals. The rats treated with curcumin exhibited a significant reduction in FBG and had increased insulin levels when compared to the negative control group rats. Another finding was in accordance with current findings that oral administration of tetrahydrocurcumin to STZ-given diabetic animals significantly increased plasma insulin and reduced plasma glucose levels [20]. Moreover, another study reported that pre-treatment with curcumin significantly decreased serum glucose levels and enhanced insulin levels [21]. Based on other natural products, related results were reported, as insulin levels and blood glucose were restored in diabetic animals by *Gymnema sylvestre* treatment [22], and *Caralluma tuberculata* caused increases in insulin levels [23]. 

In this study, the serum levels of cholesterol and TGs were increased in diabetic rats as compared to normal control rats. The treatment with curcumin significantly decreased the levels of cholesterol and TGs. A study based on curcumin reported that treatment of STZ-induced diabetic animals with curcumin reduced total cholesterol and LDL levels, indicating that curcumin reduced hyperlipidemia [24].

The rise in levels of creatinine, uric acid, and blood urea nitrogen (BUN) in the serum of diabetic rats postulates progressive renal damage, an index of changed GFR in diabetic nephropathy [25,26]. The clearance of creatinine is measured by urine and serum levels of creatinine and is a sign of functional changes in renal cells [27]. In the current study, it was observed that STZ-induced diabetic rats exhibited an increase in creatinine and urea levels. Creatinine and urea levels of STZ-induced diabetic rats treated with curcumin (50 mg/kg b.w.) were diminished. Our findings are consistent with earlier reported findings, which displayed improved renal function in diabetic rats treated with curcumin, and it was described that treatment of STZ-induced diabetic rats with curcumin reduced diabetic nephropathy with meaningful decreases in blood urea nitrogen and creatinine [28]. In addition, treating diabetic rats with curcumin caused a reduction in renal histological changes and urea nitrogen and creatinine levels, demonstrating improvements in kidney structure and function [29]. 

Oxidative stress is a state of inequality in the production and accumulation of reactive oxygen species in cells and tissues with the capability of biological systems to clear these reactive products [30]. Oxidative stress has been identified as one of the chief causes of the development of diabetes [31,32]. Thus, a molecule holding both antioxidant and hypoglycemic potential might be measured as a protective agent against diabetic nephropathy [33,34]. Diabetic rats showed an increase in MDA and a decrease in antioxidant enzyme levels as compared to normal control groups. As compared to the STZ-induced diabetic rats, curcumin enhanced the reduction in antioxidant enzymes and decreased MDA levels, suggesting that curcumin is able to reduce oxidative stress through the enhancement of antioxidant enzymes and the reduction in lipid peroxidation. Previous findings were similar to current findings, as the administration of curcumin to STZ-induced rats reduced renal dysfunction and oxidative stress [35]. Also, diabetic rats showed a substantial rise in lipid peroxidation, as identified by a noticeable rise in renal MDA levels and a decrease in glutathione levels as compared to control rats. Treatment with curcumin in diabetic rats reversed the decreased glutathione levels and resulted in a rise in lipid peroxidation [35].

Inflammation is a major factor in metabolic dysregulation. Chronic exposure to pro-inflammatory mediators arouses the activation of cytokine signaling proteins, which finally stop the initiation of insulin signaling receptors in β-cells of pancreatic islets [36,37]. However, the regulation of the inflammation process is a crucial step in the inhibition of diabetes and its complications. Curcumin treatment attenuates inflammatory marker levels as compared to untreated diabetic rats due to its potential to attenuate hyperglycemia and the inflammation process. In this regard, previous findings reported that treatment of STZ-induced diabetic rats with curcumin attenuated pro-inflammatory cytokine mRNA as well as protein levels and macrophage infiltration in renal tissue [38]. Moreover, in parallel with the present results, it was reported in these previous findings that the administration of curcumin improved antioxidant enzymes and that inflammatory cell infiltration in the liver was decreased, demonstrating reduced oxidative stress as well as inflammation [39].

A histopathological assessment of the kidney tissues of STZ-induced diabetic rats displayed various changes, such as the infiltration of inflammatory cells, congestion, and fibrosis. Treatment with curcumin significantly reduced these tissue changes, thus representing a protective role in renal damage. Previous findings reported that bioactive compounds of natural products maintain kidney tissue architecture [40]. 

Cytokines have a vital role in intercellular renal damage as well as in the production of secondary messengers, including cell adhesion molecules, acute phase proteins, and transcription factors [41]. As per the clinical data, the levels of IL-6 and IL-1β were increased in patients with diabetic nephropathy [42]. In this study, the STZ-induced diabetic group showed significantly higher expression of the IL-6 protein. However, curcumin treatment reduced the expression of IL-6. Another study based on ursolic acid reported that immunohistochemical staining revealed a rise in the expression of cytokines such as TNF-α, MCP-1, and IL-1β in diabetic nephropathy rats compared to control animals. Amazingly, the elevated expression levels of the above-mentioned proteins in diabetic nephropathy rats were attenuated by administration with ursolic acid [43]. Similarly, another study reported that IL-6 is positively expressed in STZ-treated renal tissues. On the other hand, nephropathic rats treated with chitosan-loaded *p*-coumaric acid nanoparticles (PCNPs) showed a considerable reduction in IL-6 in comparison with nephropathic rats [44]. 

TNF-α is an inflammatory cytokine, and its upregulation has been noticed in pathogenesis. This study showed the upregulation of TNF-α in the renal tissue of STZ-induced diabetic rats. Furthermore, TNF-α expression was found to be significantly decreased in the curcumin-treated group. In this regard, another study based on curcumin reported that, as compared to the control group, the diabetes mellitus group showed high positive cells. After treatment with curcumin, the expression of this inflammatory protein decreased significantly [45].

## 4. Materials and Methods

### 4.1. Chemicals

Streptozotocin (STZ) and curcumin were purchased from Sigma-Aldrich Inc., St. Louis, MO, USA. The antioxidant enzyme kits including catalase, glutathione, and superoxide dismutase were acquired from Abcam, Cambridge, UK. The inflammatory markers kits for TNF-α, IL-6 and IL-1β were also obtained from Abcam, UK. Antioxidant enzyme and myeloperoxidase kits were purchased from Abcam, UK. For fibrosis evaluation, trichrome stain and a Sirius red kit were purchased from the same company. IL-6 and TNF-α monoclonal antibodies and the Specific HRP/DAB Detection IHC kit were acquired from Abcam, United Kingdom. All supportive chemicals used in this study were of analytical grade and purchased from a local vendor.

### 4.2. Animals and Treatment

A total of 32 male albino rats, weighing 160–210 g, were purchased from the animal house of King Saud University, Saudi Arabia. All rats were kept under normal research laboratory conditions with a 12 h light/dark cycle at a temperature of 23 ± 2 °C during the study. Rats were fed with normal rat chow as well as tap water ad libitum throughout the experiment. All methodology was approved by the Control and Regulation of Experiments on Animals, CAMS, at Qassim University (ethical committee no. 31711J1-2022-1-3-C). 

This study used STZ to induce type 2 diabetes mellitus (T2DM) in rats with a single dose of a streptozotocin (55 mg/kg b.w.) [46] solution prepared in 50 mM citrate buffer. Curcumin (50 mg/kg b.w.) [47,48] was prepared in a dimethyl sulfoxide (1.0%) solution and administered orally by gavage to the treatment animals. After 2 days of STZ injection, blood glucose was measured, and rats with a glucose level >200 mg/dl were considered diabetic animals and considered as the diabetic group. Curcumin was given thrice weekly after one week of diabetes induction and continued for eight consecutive weeks. Glibenclamide (5 mg/kg b.w.) was given to rats and considered a positive control.

Rats were divided into 4 different groups, with 8 rats per group:

- Group I: rats with free access to rat pellets and orally administered saline as a placebo for 8 weeks, considered as the normal control (C) group.
- Group II: STZ-induced diabetic rats at 55 mg/kg b.w., considered as the negative control (NC) group.
- Group III: diabetic rats administered curcumin orally (50 mg/kg/day) [47,48] for 8 weeks, considered as the curcumin treatment (CrT) group.
- Group IV: diabetic rats treated with glibenclamide (5 mg/kg b.w.) [49] as a standard drug for 8 weeks, considered as the positive control (PC) group.

### 4.3. Effect of Curcumin on Oral Glucose Tolerance Tests

As described by the previous method, an oral glucose tolerance test (OGTT) was executed [50] after constant treatment for 8 weeks. Briefly, fasting rats were orally given glucose (3 g/kg b.w.). Blood samples were collected from the tail vein at different intervals: 0 min, 30, 60, 90, and 120 min after glucose administration. Blood glucose levels were examined by using a glucometer.

### 4.4. Measurement of Fasting Blood Glucose and Insulin Level

After the completion of the treatment plan, all the animals were sacrificed, and blood samples were collected for the estimation of glucose and insulin levels by using specific kits.

### 4.5. Total Cholesterol and Triglyceride (TG) Measurement

The total cholesterol (TC) and triglycerides (TGs) were measured using the colorimetric method. The absorbance of the colored solution was evaluated at 546 nm, and the results were interpreted accordingly.

### 4.6. Determination of Serum Urea and Creatinine Level

Blood was collected and centrifuged at 3000× *g* for 20 min. The obtained serum was measured from all the experimental animals for urea and creatinine levels, the absorbance was measured, and the results were interpreted accordingly.

### 4.7. Measurement of Malondialdehyde (MDA) 

The determination of malondialdehyde (MDA) content was estimated through the thiobarbituric acid (TBA) method for a non-enzymatic oxidative state for lipid peroxidation [51]. Lipid peroxidation was measured by estimating the malondialdehyde (MDA) concentration, which was evaluated by the absorbance of color development with thiobarbituric acid. The absorbance of the resulting product was estimated at 532 nm, and the results are expressed in nmol/g [52].

### 4.8. Determination of Antioxidant Enzyme (SOD, GST, and CAT) Levels

Renal tissue was obtained from different experimental groups of rats, and all tissues were stored in PBS. All the samples were homogenized and centrifuged at 1100× *g* for 15 min. The antioxidant enzyme (CAT, SOD, and GST) levels were measured in different experimental groups using the colorimetric method with commercial kits (Abcam, UK). The absorbance of the resultant product was estimated at different wavelengths [52] as per the manufacturer’s instructions, and the results were consequently interpreted.

### 4.9. Assessment of Inflammatory Cytokines (IL-6, IL-1β, and TNF-α)

The Enzyme-Linked Immunosorbent Assay (ELISA) test was run to quantify the evaluation of inflammatory markers. The inflammatory markers, including IL-6, IL-1β, and TNF-α, were measured using an ELISA Kit (Abcam, Cambridge, UK) according to the manufacturer’s protocol, and the obtained results were expressed as pg/mL [53] accordingly.

### 4.10. Histopathological Examination of Renal Tissues

Kidney tissues were taken and immediately fixed in 10% buffered formalin. Then, tissues were processed, followed by dehydration using a series of graded alcohols (Leica automated tissue processor, Leica TP1020), Nussloch, Germany and embedded in paraffin wax (Leica embedding unit, Leica EG1160) to make paraffin-embedded blocks. Kidney sections (5 μm thick) were cut using a rotatory microtome (Leica RM2125). Hematoxylin and eosin staining was performed as per a previously described protocol [54], and independent pathologists examined the slides in a blinded manner. Stained slides were examined under a light microscope (Leica) at 100× magnification. The photographs were taken by a Leica Digital camera, DMD108, and the findings were consequently construed.

### 4.11. Fibrosis Evaluation Using Masson Trichrome and Sirius Red Staining 

Masson’s trichrome staining kit was used to measure collagen fibers. Briefly, kidney sections were deparaffinized and hydrated properly. The manufacturer’s instructions were followed to complete the staining procedures. Finally, all slides were cleared by xylene and mounted using D.P.X. Sections that underwent Masson trichrome and Sirius red staining were evaluated under a light microscope (Leica) using 100× magnification. Photographs (Leica Digital camera DMD108) were taken and findings were consequently construed.

### 4.12. Expressional Evaluation of IL-6 and TNF-α Proteins Using Immunohistochemistry Staining

Kidney sections were used for immunohistochemical staining to evaluate the expression pattern of IL-6 and TNF-α proteins, as previously described [55,56]. Briefly, sections were deparaffinized and dehydrated, and antigen retrieval was performed in citrate buffer (pH 6.0) for 22 min. After washing in PBS, the slides were incubated in blocking buffer for 10 min. After that, the sections were incubated with IL-6 and TNF-α primary antibodies at 4 °C overnight. The slides were washed away with phosphate buffer solution and incubated with secondary antibodies in a moistened atmosphere for 30 min. Finally, the slides were incubated with DAB, and sections were dehydrated. Sections were mounted with D.P.X and covered with coverslips. A light Leica microscope (Leica, DM2500) using under 100× magnification was used, photographs (Leica Digital camera, DMD108) were taken, and findings were consequently construed.

### 4.13. Quantification of IHC

IL-6 and TNF-α protein expression were measured based on cytoplasmic staining. A pathologist blinded to the experimental group using a light microscope assessed the quantification of positively cytoplasmic-stained renal cells. Five different areas (a total number of 500 cells) of each section were selected, and the number of stained cells in each region was calculated. The cells were scored as negative (if less than 5% positivity) or positive (if more than 5% of cells showed positivity) and intensity of staining as negative, moderate, or strong. The percentage of positive cells was categorized as follows: 0 (negative), 1 (10–15%), 2 (16–30%), 3 (31–50%), and 4 (more than 50%).

### 4.14. Statistical Analysis

All the data obtained from these results were evaluated statistically as means ± standard error of mean (SEM). One-way analysis of variance (ANOVA) was used for calculations in multiple groups. A *p* < 0.05 was measured as statistically significant.

## 5. Conclusions

The current study was carried out to measure the anti-diabetic potential of curcumin through physiological, biochemical, and histopathological studies. In the physiological and biochemical studies, it was found that curcumin decreases glucose, creatinine, urea, and inflammatory markers and increases antioxidant enzyme levels. In addition, the histopathological findings revealed that curcumin plays a significant role in the maintenance of renal tissue architecture through the reduction in all pathological changes.

This study showed that curcumin has a vital role in the regulation of the expression pattern of the IL-6 protein and fibrosis. Based on biochemical and histopathological findings, this study delivers a scientific suggestion that curcumin could be a suitable remedy in the management of diabetes mellitus. The novelty of the current study is that curcumin showed anti-fibrotic potential by reducing collagen fiber deposition. However, more detailed studies based on molecular mechanisms are needed to know the mechanism of action of this compound in diabetes prevention and management.

## Data Availability

The data used to support the findings of this study are included within the article.
